# Choosing between staying at home or moving: A systematic review of factors influencing housing decisions among frail older adults

**DOI:** 10.1371/journal.pone.0189266

**Published:** 2018-01-02

**Authors:** Noémie Roy, Roxanne Dubé, Carole Després, Adriana Freitas, France Légaré

**Affiliations:** 1 Interdisciplinary Research Group on Suburbs (GIRBa), Laval University, Quebec, Qc, Canada; 2 School of Architecture, Laval University, Quebec, Qc, Canada; 3 Laval University Primary Care Research Centre (CERSSPL-UL), Quebec, Qc, Canada; 4 Department of Family Medicine and Emergency Medicine, Laval University, Quebec, Qc, Canada; TNO, NETHERLANDS

## Abstract

**Background:**

Most older adults wish to stay at home during their late life years, but physical disabilities and cognitive impairment may force them to face a housing decision. However, they lack relevant information to make informed value-based housing decisions. Consequently, we sought to identify the sets of factors influencing the housing decision-making of older adults.

**Methods:**

We performed a systematic literature search for studies evaluating any factors influencing the housing decisions among older adults over 65 years old without cognitive disabilities. Primary research from any study design reported after 1990 in a peer-reviewed journal, a book chapter or an evaluated doctoral thesis and written in English, French or Spanish were eligible. We extracted the main study characteristics, the participant characteristics and any factors reported as associated with the housing decision. We conducted a qualitative thematic analysis from the perspective of the meaning and experience of home.

**Results:**

The search resulted in 660 titles (after duplicate removal) from which 86 studies were kept for analysis. One study out of five reported exclusively on frail older adults (n = 17) and two on adults over 75 years old. Overall, a total of 88 factors were identified, of which 71 seem to have an influence on the housing decision-making of older adults, although the influence of 19 of them remains uncertain due to discrepancies between research methodologies. No conclusion was made regarding 12 additional factors due to lack of evidence.

**Conclusion:**

A wealth of factors were found to influence housing decisions among older adults. However, very few of them have been studied extensively. Our results highlight the importance of interdisciplinary teamwork to study the influence of a broader range of factors as a whole. These results will help older adults make the best possible housing decision based on their unique situation and values.

## Introduction

The proportion of older adults around the world is increasing dramatically. By 2050, the number of adults aged 60 years and over will nearly double and countries in Europe and North America will see the proportion of older adults increase by 30% [1]. In Canada, estimates from 2015 show that for the first time, there were more older adults in the country than children under 15 [2] and the number of adults aged 65 and older will represent more than 25% of the population by 2036, with 32% of them being over 80 years old [3]. As their age increases, older adults inevitably experience a progressive loss of their capacities and autonomy. Indeed, 57% of Canadians aged 85 and older report functional limitations, compared to 12% of adults between the age of 65 and 74 [4]. In addition, 30% of Canadians aged 75 years and over are receiving home care services [5]. Functional limitations among older adults will increase the pressure on healthcare services, social services and on the provision of housing as aging Canadians consider whether to stay at home or relocate.

Choosing between staying at home and moving to a more supportive environment is a complex decision for older adults facing autonomy loss. Most of them would prefer to stay in their home [6, 7]. Indeed, 90% of Canadians aged 65 years old and over still live in their homes. Two-thirds of them live in a private house [8] and over 70% have not moved in the past five years [9]. The residential mobility rate of older Canadians even decreases with age: adults of 85 years or older living in private dwellings are 30% less likely to have moved than adults aged 65–69 years old [10]. However, half of adults aged 85 years or older living at home rely on caregivers or on home care services to help them perform their daily activities [5]. When this help becomes insufficient, frail older adults are likely to consider their housing options: either stay in their home and adapt it to their needs, or move to an already adapted dwelling, with or without additional care.

Over the years, studies investigating housing decisions have used several conceptual models. Three main theory families on living arrangements in old age have received more extensive empirical testing than the others [11]: migration theory [12–14], environmental press theories [15] and health behaviors theories [16]. However, none of these theories considered the residential experiences of older adults as well as the social and emotional meanings attached to these experiences. The role these factors play in older adults’ decision-making about housing options is therefore still largely unspecified [11]. A framework proposed by Després and Lord [17] encompasses these lesser explored factors, looking at older adults’ housing decisions through a new lens, the meta-concept of *home*. They suggest six main dimensions that best account for the meanings and experiences of home (Table 1).

**Table 1:** The experience and the meaning of home, by Després and Lord (2005).

*Home* as …		
**Psychological dimensions** Mirror of the self Place to personalize Personal control Physical and psychological security Physiological and physical comfort	**Economic dimensions** Ownership Financial investment Savings and inheritance Affordable housing	**Temporal dimensions** Familiar setting Attachment and memories
		**Space-time dimensions** Anchor Center of daily life Territory of mobility Settlement-identity Proximity and accessibility
	**Material dimensions** Network of urban places Urban territory Services and commercial facilities Nature and greenery Housing type Space around the house Safety and universal accessibility Personal belongings	
**Social dimensions** Locus of socialization Privacy and refuge Indicator of social status Desirable social composition Access to human resources		



To date, many factors have been taken into account in research on older adults’ housing decisions, including health and social factors. However, not all potential factors have been identified, especially those related to the built environment and what it represents for older adults. Thus, our objectives were: 1) to identify all the factors that influence decision-making about housing options among older adults with loss of autonomy; 2) to classify them according to a new adapted framework that combines health, safety and functional autonomy factors with those related to the meaning and experience of home; and 3) to observe which factors had an observed effect in the research and which need further investigation.

## Methods

### Study design

We conducted a systematic review to evaluate: What are the factors that influence older adults without cognitive disabilities when faced with a housing decision? Our specific questions were: 1) besides factors related to health and functional autonomy identified as influencing the housing decision of older adults, what is the role of factors related to their experience of home and the meanings they attached to it?; 2) in what other countries are studies of the factors influencing the housing decisions of older adults taking place, and in what research disciplines?

We refer to “staying at home” as the older adult staying in their current dwelling where they feel at home and to which they attached social and emotional meanings. Staying at home can be achieved alone or with a caregiver, it can involve home care services, home modifications, or neither of these.

### Information sources

The search strategy was developed by the authors in consultation with an information specialist. Searches were conducted from database inception until the end of February 2015. Our literature search used the keywords “older adults”, “frail”, “housing decision”, “housing relocation” and “factors”. As we wanted to explore literature from diverse disciplines, we searched for primary studies in AgeLine, ERIC, PubMed, Taylor & Francis and Web of Science.

Only the database searches in AgeLine and Taylor & Francis were limited. In AgeLine, we included studies on older adults without dementia (“NOT dementia”). In Taylor & Francis, we included studies on older adults without dementia or mental disability (“NOT ‘mental disability’”, “NOT dementia”) and we excluded studies focusing on politics or drugs (“NOT politics”, “NOT drugs”). These restrictions were to clarify the search and to limit the vast spectrum obtained with the main strategy (S1 Appendix. Search strategy example). In addition to our database search, we also invited team members (e.g. experts in health sciences and the built environment) to inform us of any other potentially relevant study.

### Eligibility criteria

All or some participants in the included studies had to be aged over 65 years and we excluded those with cognitive disabilities. If the age range of participants was not specified, we included studies in which participants’ mean age was over 65 years or participants who were recruited in housing designed for older adults, with or without additional care. We included studies of any kind of intervention aimed at reporting or measuring factors influencing the housing decision. We included studies both with and without comparison groups. Study outcomes could be any objective or subjective measures of factors influencing housing decisions as reported by experts or as self-reported by participants. There was no restriction on study design. We included all articles in peer-reviewed journals, book chapters in books with editorial committees or doctoral theses with thesis committees. We excluded studies published before 1990 because the important developments in environmental gerontology and around the meta-concept of *home* occurred after that date [18].

### Study selection

Two of the authors (NR and RD) combined search results and independently checked for duplicates. A pre-test screening using a Kappa k calculation was performed on 60 randomly selected titles and abstracts to check concordance between the two authors. The coefficient of Kappa k was 0.8691, corresponding to “excellent” agreement between the authors. This pre-test allowed the authors to discuss the abstracts they disagreed about and to adjust their screening accordingly. Then they individually evaluated the remaining titles and abstracts and discussed in person all studies for which inclusion and exclusion criteria were not clear from the title or abstract. Any remaining disagreements were resolved through discussion with a third author (CD). Full-text copies of all studies that might be relevant and had not been excluded through screening were retrieved. All full-texts were reviewed by the authors (NR and RD) and again discussed to check agreement that they met the pre-established inclusion and exclusion criteria.

### Data collection process

Two authors (NR and RD) extracted data independently from eligible studies using a data extraction sheet. General characteristics (e.g. publication year, country of study, authors’ discipline of study retrieved from their curriculum vitae), study characteristics (e.g. study objectives, study design, data collection, nature of reported issues), participants characteristics (minimum age included, mean age, sample size, autonomy level, type of dwelling and neighborhood, tenure status) were extracted, as well as factors reported as associated with the decision to relocate or not, whether the factors were identified as statistically significant or not in quantitative study designs, or narratively reported in qualitative study designs. The extraction grid was inspired by the framework proposed by Després and Lord [17] to which was added a fifth dimension to include the socioeconomic and health-related factors of influence on the experience and meaning of home. After discussion with team members, the space-time dimension and the temporal dimension were also combined into one *time and space-time related dimension* due to their similarities, the psychological dimension was extended to include psychosocial factors, and the material dimension became the *built and natural environment dimension*. The authors subsequently classified all factors influencing housing decisions, as extracted from the studies, into the resulting six dimensions of the new framework. Each author (NR and RD) reviewed the other’s extraction and resolved doubts or disagreements. Any remaining disagreements were adjudicated by CD.

### Quality appraisal

The authors (NR and RD) appraised the quality of studies using the Mixed Methods Appraisal Tool (MMAT) [19]. The MMAT is a validated checklist for appraising the quality of quantitative, qualitative, and mixed methods-studies included in systematic reviews [20]. For quantitative randomized controlled studies, we assessed randomization, allocation concealment or blinding, completeness of outcome data, and withdrawal/drop-out rates. For quantitative non-randomized studies, we assessed selection bias, appropriateness of measurements, comparability of groups, completeness of outcome data and response or follow-up rates. For quantitative descriptive studies, we assessed sampling strategies, sample representativeness, appropriateness of measurements and response rates. In qualitative studies, we assessed the relevance of the data sources, the relevance of the analysis process, context consideration and consideration of researcher influence. In mixed-methods studies, we assessed the quality of both qualitative and quantitative components.

After discussing their appraisals, the two authors (NR and RD) resolved any remaining doubts or disagreements through discussion with a third author (CD). Missing information was sought either by searching the website of the research project (if available) or contacting the authors.

### Synthesis of results

Given the high level of methodological heterogeneity across studies, the authors conducted a qualitative synthesis of the studies. They also compared the results according to their study design (qualitative, quantitative and mixed method). The factors were classified by the level of agreement between studies that found an effect on the housing decision. Factors studied by fewer than three quantitative studies or fewer than five studies of any design method were treated as exploratory factors. The level of agreement between studies was therefore not calculated.

## Results

### Study selection

**Fig 1:**
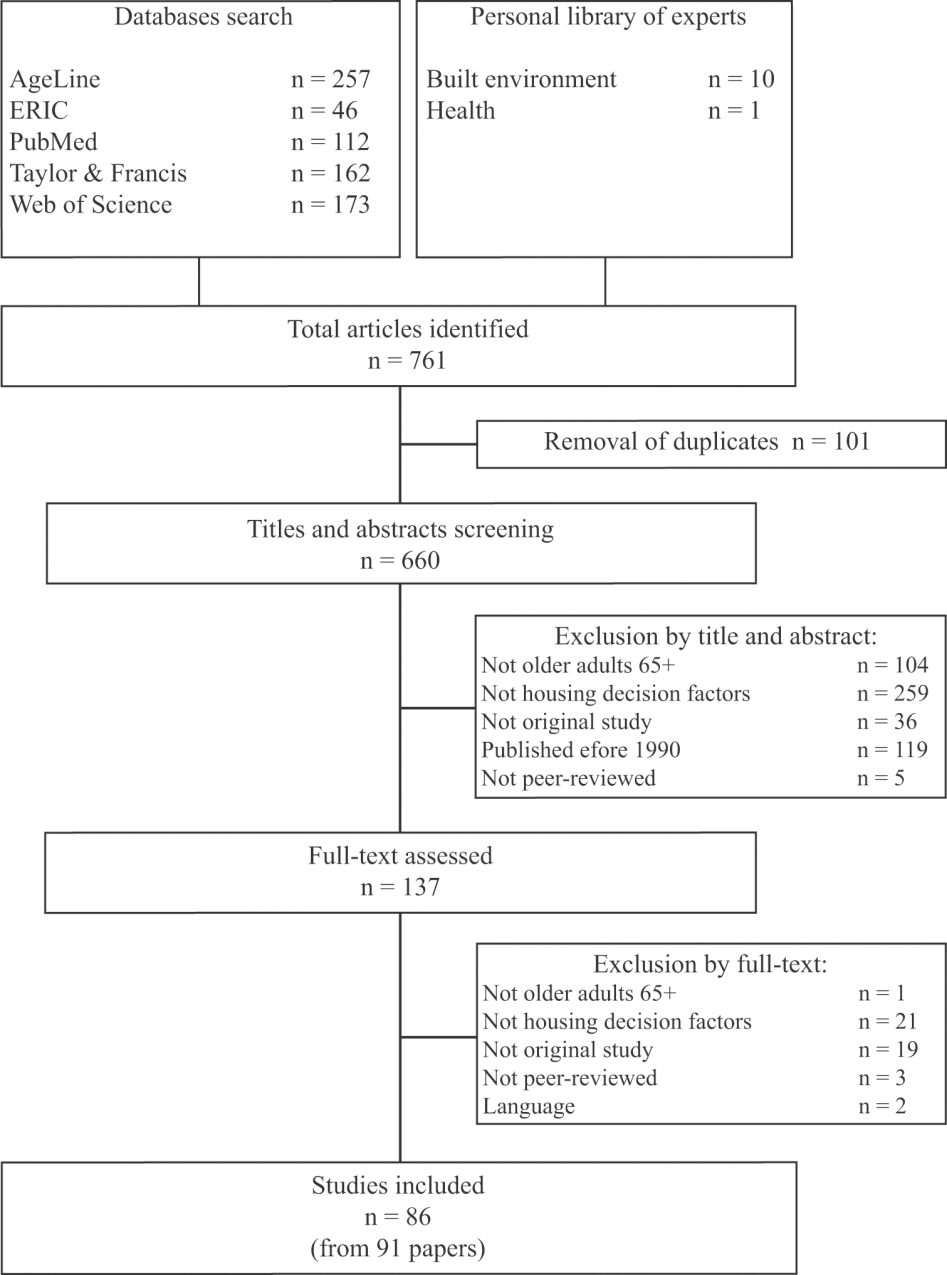
Flow chart.

Of 761 potential studies investigating the factors influencing housing decisions that were retained for this review, 750 were identified through the database search and 11 through team members. After removing duplicates, 660 studies were reviewed for eligibility. Eighty-six independent studies, described in 91 publications, met all eligibility criteria and were kept for analysis (Fig 1). As three research studies were described in more than one publication [21–28], all publications that reported on them are cited together when referring to these studies.

### Study characteristics

Table 2 presents an overview of the extracted studies’ characteristics. All of them were published in English except for two, which were in French [27, 29]. A total of 74 studies were published in peer-reviewed journals [10, 21–28, 30–99]; nine more were retrieved from doctoral theses [100–108] and three others from book chapters reviewed by editorial committees [29, 109, 110]. Over half were published after 2005 (n = 50) [10, 21–23, 25–39, 44, 46–48, 51, 53, 55–58, 60–62, 64–66, 68, 69, 71, 74, 77, 78, 80, 81, 84–86, 88, 90–92, 96, 98, 99, 106]. Over three quarters of independent studies were conducted in the USA (n = 48) [21, 24, 31, 33, 36–38, 40–43, 47, 51, 54, 55, 57, 58, 62, 63, 65–67, 70, 71, 73, 75, 77, 80, 81, 83, 84, 87–90, 93–95, 97, 100–105, 107–110], Australia (n = 10) [34, 35, 39, 44, 49, 52, 72, 86, 91, 96] and Canada (n = 9) [10, 26, 27, 46, 59, 68, 78, 85, 98, 106]. Four studies were conducted across more than one country: two in Europe [22, 23, 25, 28, 30], one in Asia [108] and one in the USA and Germany [82].

**Table 2:** Study characteristics addressing the housing decision factors in alphabetic order (n = 86, described in 91 publications).

Independent studies								
Authors	Ref #	Year	Location	Authors field of study	Aim/purpose	Method	Participants (n)	Study quality
Angelini and Laferrere	30	2012	Austria-Belgium-Denmark-France-Germany-Greece-Italy-Netherlands- Spain-Sweden-Switzerland	Economy, statistics	To analyze the residential mobility choices of older adults and the factors influencing them in the evolution of their housing consumption and their investment in a home.	Quantitative study, incidence/prevalence study, longitudinal	17750 (aged≥ 50)	H
Ball *et al*.	31	2009	USA	Medicine, administration, sociology, gerontology (2)	To examine how race and class influence decisions to move to assisted living facilities.	Qualitative study, grounded theory	60 older adults (aged≥ 65) 43 family members and friends 12 administrators	H
Battisto*	100	2004	USA	Architecture	To explore the factors common to older adults who choose to stay at home versus moving, and understand the environmental context and the decision-making process that are associated with aging in place.	Mixed method, sequential explanatory, longitudinal	8222 (aged ≥70, mean age 85) Subsample : 20 (aged ≥70, mean age 79)	H
BÄUmker *et al*.	32	2012	UK	Health economy, psychology (2), statistics, public politics, social science	To identify the factors motivating older people to move to extra care housing, their expectations of living in this new environment, and whether these differ for people moving to smaller or larger retirement communities.	Quantitative study, case series	1439 (aged≥ 55, mean 77)	H
Bekhet *et al*.	33	2009	USA	Nursing (2), medicine	To understand why older adults move to retirement communities and what living in retirement communities is like from their perspective.	Qualitative study, phenomenology	104 (aged≥ 65, mean age 82)	M
Bohle *et al*.	34	2014	Australia	Psychology, economy, specialized education, philosophy	To explore influences on the housing choices of retirees, their attitudes towards their current homes and their perceptions of the alternative type of housing available.	Qualitative study, grounded theory	81 (aged≥ 55, mean age 76)	H
Boldy *et al*.	35	2011	Australia	Psychology (3), physiotherapy, architecture	To identify the key push and pull factors that influence older adults to move from their home, think about moving from their home and to stay in their home.	Mixed method, sequential explanatory	3050 (aged ≥ 50) Subsample 39 (aged ≥ 50)	M
Buurman *et al*.	36	2014	USA	Medicine (5), theology, nursing	To describe the rates of residential relocations over the course of 10.5 years and evaluate differences in these relocation rates according to gender and deceased status.	Quantitative study, prospective, longitudinal	754 (aged≥ 70, mean age 78,4)	H
Cai *et al*.	37	2009	USA	Statistics, social work, health administration	To identify key factors associated with long-stay nursing home admission among older adults.	Quantitative study, incidence/prevalence study, longitudinal	5980 (aged≥ 65, mean age 78)	H
Caro *et al*.	38	2012	USA	Sociology, gerontology (2), engineering, economy (3)	To determine how five distinct dimensions—functional status, features of current housing, social networks, features of retirement communities, and financial considerations—affect decisions to relocate to a retirement community.	Quantitative study, incidence/prevalence study	215 older adults 51 adult children 51 parents of adult children (respective median age : 73, 62 and 84)	M
Cheek *et al*.	39	2005	Australia	Nursing (2), education	To explore and describe the factors influencing the decisions of older people living in the community in independent living units to enter the acute care system.	Qualitative method, case study	31 older adults (aged≥ 65) 10 family members 14 focus groups	H
Choi	40	2003	USA	Social work	To analyze elderly parents’ and their children’s characteristics associated with the transitions into and out of intergenerational coresidence.	Quantitative study, incidence/prevalence study, longitudinal	3648 (aged≥ 65, mean age 79)	H
Clark *et al*.	41	1996	USA	Economy (3)	To investigate the impact of locational and individual characteristics upon interstate retiree migration, particularly in state-level public policy variables.	Quantitative method, case series	4105 (aged ≥ 55)	H
Clark and Davies	42	1990	USA	Geography (2)	To analyze the effects of economic aspects on older adults’ relocation in the context of the larger issues of migration and population movements.	Quantitative method, case series, longitudinal	32 073 (aged ≥ 55)	M
Clark and White	43	1990	USA	Geography, ND	To clarify the importance of economic factors rather than dwelling or housing characteristics on older adults’ relocation within the city.	Quantitative method, Case series	ND (aged ≥65)	H
Connel and Stanford^§^	109	1997	USA	Health science, architecture	To identify residential needs of older adults with limitations and describe housing adaptations meeting those needs based on six contextual elements: the consumer, the family, commercial housing, technology, service agencies, and legislation.	Qualitative method, case study, longitudinal	17 (aged 50–80)	M
Crisp *et al*.	44	2013	Australia	Psychology (4)	To identify factors that older adults find encouraging or discouraging about the prospect of relocation to a retirement village.	Quantitative study, incidence/prevalence study	517 (aged≥ 55, mean age 65)	M
Disney *et al*.	45	2002	UK	Economy (3)	To analyze the effect of changes in household housing wealth, housing costs and saving in financial assets on housing decision of older owner.	Quantitative study, case series, longitudinal	2500 (aged 55–69)	M
Dupuis-Blanchard	46	2007	Canada	Nursing	To describe women’s experiences of relocating to an apartment building for older adults and the factors that influence relocation.	Qualitative study, grounded theory	11 (aged≥ 65)	H
Edmonston and Lee	10	2014	Canada	Sociology (2)	To identify trends in the residential mobility of older adults and to offer explanations for possible changes in older adults’ mobility trends over time.	Quantitative study, incidence/prevalence study, longitudinal	502 075 (aged≥ 65)	H
Erickson *et al*.^††^	21	2006	USA	Human development, psychology (2), sociology	To examine the relationship of health, social and financial resources, housing characteristics and residential satisfaction to the moving plans of older adults and how moving plans are related to actual moves.	Quantitative study, Incidence/prevalence, longitudinal	333 (aged ≥60, mean age 72)	M
Ewen and Chahal	47	2013	USA	Psychology, gerontology	To identify the push-pull factors associated with moving into congregate older adults housing, as well as to investigate the decision-making processes.	Mixed method, sequential explanatory	26 (mean age 76)	H
Fonad *et al*.	48	2006	Sweden	Neurosciences (2), nursing (2)	To investigate the experience of safety and security in their previous dwelling of older adults who recently moved to a retirement home.	Qualitative study, case study	57 (aged≥ 65, mean age 84)	H
Fornaro*	101	2004	USA	Urban planner	To determine if neighborhood satisfaction is a factor in the decisions made by older adults to relocate from their existing home and neighborhood.	Mixed method, triangulation	46 (aged ≥ 55)	M
Gardner	49	1994	Australia	Social worker	To investigate how housing attainment in old age interacts with life span vulnerabilities to influence the decision to move to retirement village.	Qualitative method, case study	80 (mean age 73)	M
Glaser *et al*.	50	2003	England	Sociology, demography, computer sciences	To examine changes in the proportion of older widowed and divorced women moving from ‘independent’ to private and institutional ‘supported’ housing.	Quantitative study, Incidence/prevalence, longitudinal	18 786 (aged ≥65)	M
Granbom *et al*.^†^	22	2014	Germany-Sweden	Occupational therapy (4), gerontology, psychology	To explore the process of residential reasoning and how it changes over time among very old people.	Qualitative study, narrative, longitudinal	16 (aged 80–89)	H
Granbom *et al*.^†^	23	2014	Sweden	Occupational therapy (4), epidemiology	To identify which aspects of housing and health predict relocation to ordinary or special housing among very old people.	Quantitative study, incidence/prevalence study, longitudinal	384 (aged 80–89, mean age 85)	M
Groger and Kinney	51	2006	USA	Psychology, anthropology	To describe older adults’ reasons for moving into continuing care retirement communities and their perceptions of the trade-off or anticipated gains and losses inherent in the move.	Qualitative study, case study	20 (aged≥ 65, mean age 75)	H
Groves and Wilson	52	1992	Australia	Psychology (2)	To assess those factors which have the greatest influence upon housing choices made by older adults.	Quantitative method, Case report	102 (aged ≥ 60, mean age 73.4)	M
Hansen and Gottschalk	53	2006	Denmark	Economy, engineering	To determine which factors influence older people’s considerations about moving house and which influence actual mobility, and on this basis to uncover what factors further or hinder considerations about moving house.	Quantitative study, incidence/prevalence, longitudinal	5260 (aged 52–77)	H
Hersch *et al*.	54	2004	USA	Occupational therapy (9)	To examine the relocation pathways of older adults and to identify adaptive challenges and the strategies used to address them.	Mixed method, triangulation	10 (aged≥ 50)	H
Hong and Chen	55	2009	USA	Social work (2)	To assess the impact of relocation, residential type, and individual lifestyle factors on the structure of health status overtime.	Quantitative study, case series, longitudinal	5294 (aged≥ 70, mean 75)	H
Hui and Yu	56	2009	China	Economy, social sciences	To investigate how various attributes, from residential to institutional, influence the housing satisfaction of older adults, and thus their likelihood of residential relocation.	Quantitative study, cross-sectional	332 632 (aged ≥50)	H
Jennings *et al*.	57	2014	USA	Social work (2), social worker+anthropology	To examine the reasons for older adults’ transition to continuing care retirement communities, condominiums, or smaller homes, rather than collocate with kin.	Qualitative study, ethnography	81 older adults (aged≥ 65) 49 family members	H
Johnson and Bibbo	58	2014	USA	Gerontology, psychology	To uncover the meaning of home for older adults in nursing homes shortly following the relocation and approximately two months later.	Qualitative study, phenomenology	8 (aged≥ 65, mean age 81)	H
Jones	59	1997	Canada	Economy	To estimate the tenure transition likelihood of older households who are homeowners.	Quantitative method, case series	120 (aged ≥ 55)	H
Jörg *et al*.	60	2006	Sweden	Psychology, public health, social sciences, education	To determine what older adults, needs assessors, and agency factors explain variation in decision making needs of assessors concerning older adults requesting admission to long-term care housing.	Quantitative study, incidence/prevalence study	214 needs assessors Older adults’ vignette assessed aged 73 or 85	H
Jorgensen *et al*.	61	2009	New Zealand	Physiotherapy, social sciences, public health, psychology, nursing+psychology	To investigate why older adults with high support needs entered residential care and who made that decision.	Mixed method, triangulation, longitudinal	144 older adults (aged≥ 65); 47 unpaid caregivers; 12 service co-ordinators; 4 multidisciplinary team members	M
Jungers	62	2010	USA	Orientation	To describe older adults' experiences of a late-life residential relocation from a home to a long-term health care setting.	Qualitative study, narrative	14 (aged≥ 75, mean age 85)	H
Kampfe	63	2002	USA	Rehabilitation	To examine the degree to which older adults perceived their moves to be important, controllable, stressful, disruptive, and positive.	Quantitative study, case series	102 (aged≥ 65, mean age 83)	H
Keese	64	2012	Germany	Economy	To investigate housing consumption and home ownership in the elderly.	Quantitative study, case series, longitudinal	161 235 households Comparison between age groups (from <30 to ≥80)	H
Kemp	65	2008	USA	Sociology	To determine the pathways leading couples to reside together in assisted living facilities.	Qualitative study, Phenomenology	20 (aged 66–94, mean age 86) 10 adult children	H
Kim *et al*.	66	2014	USA	Psychology, architecture, engineering, neuroscience	To examine factors of home modification in frail older adults and their informal caregivers for improving health care at home.	Quantitative study, incidence/prevalence study	737 (aged≥ 65, mean age 82)	H
Kim*	102	2002	USA	Architecture	To examine the features that make residents feel “at home” in assisted living facilities and to suggest further policy and design guidelines.	Qualitative study, case study	25 (aged≥ 65, mean age 82)	H
Knotts*	103	2003	USA	Occupational therapy	To investigate the meaning of place transitions or relocations to older adult in terms of how it affected their quality of life.	Qualitative method, phenomenology	12 (aged ≥70)	H
Koenig and Cunningham	67	2001	USA	Psychology (2)	To identify the reasons why individuals relocate and whether movers differ from nonmovers on demographic, social, and personality factors.	Quantitative study, case series	100 (aged 34–93) Comparison between 3 age groups (mean age 39, 62, 75)	M
Krout *et al*. ^††^	24	2002	USA	Sociology (2), physiotherapy, psychology (2)	To examine the reasons given by older adults for relocation to a continuing care retirement community.	Quantitative study, case series	91 (aged 65–95)	H
Lai	68	2005	Canada	Social work	To examine preferred living arrangements of Chinese-Canadians’ older adults.	Quantitative study, case series	2272 (aged≥ 55, mean age 70)	H
Lee*	104	2003	USA	Design	To identify factors prompting the decision of older households to move to recently built older adults co-op or rental housing, and factors affecting the satisfaction with previous and current residential environments.	Quantitative method, cross-sectional	280 (aged≥ 55)	M
Leesson	69	2006	Denmark	Demography	To examine the attitude and expectations of older adults concerning housing.	Quantitative study, incidence/prevalence, longitudinal	3903 (aged 40–79)	M
Leith	70	2004	USA	Social work	To explore the meaning of home for older women living in a congregate housing complex who have been more or less successful in making it a home.	Qualitative method, phenomenology	20 (aged 63–91, mean age 77.95)	H
Löfqvist *et al*. ^†^	25	2013	Germany-Sweden	Occupational therapy (4), gerontology, psychology	To explore how very old adults reflect upon relocation and aging in place.	Qualitative study, qualitative description	80 (aged 80–89, mean age 85)	H
Lord *et al*.^‡^	26	2011	Canada	Urban planner, architecture, psychology	To understand how older adults stay mobile in their home and their neighborhood faced with the need to adapt to reduced autonomy and mobility over time.	Qualitative method, narrative, longitudinal	22 (aged 62–89)	H
Lord *et al*.^‡^	27	2009	Canada	Urban planner, engineering, geography	To compare the practices and meanings of daily mobility of older adults belonging to three age-groups and currently residing in postwar suburbs.	Mixed method, sequential explanatory, longitudinal	87 (aged 55–82)	M
Luborsky *et al*.	71	2011	USA	Anthropology (2), occupational therapy	To examine how key contours of the experiences of place during residential downsizing are infused with unexpectedly heightened awareness and cultivation of older adults’ sense of place in multiple timeframes.	Qualitative method, narrative	40 (aged≥60, mean age 74)	M
McKenzie	72	2002	Australia	Geography	To highlight the significant emotional impact which car relinquishment can have for older people and on their relocation decision.	Qualitative study, qualitative description	16 (aged ≥ 60)	M
Megbolugbe	73	1999	USA	Administration (3)	To compare housing decisions and tenure transitions between older men and older women.	Quantitative study, case report	1000 (aged≥ 55)	H
Millage*	105	1990	USA	Economy	To study the influences on and content of communication between retirement housing providers and retirees who are their potential customers.	Qualitative method, case study	120 (aged ≥ 60)	H
Motevasel et al.	74	2006	Sweden	Social work	To explore older adults’ reasons for moving to private senior housing and to identify the differences between them and residents of tenant-owned housing cooperatives.	Qualitative study, qualitative description	28 (aged≥ 55)	H
Mutchler and Burr	75	2003	USA	Sociology (2)	To examine the effects of housing market conditions on the living arrangements of non-Hispanic White and African American older adults.	Quantitative study, case series	178 006 unmarried older adults 96 371 couples (aged≥ 60)	H
Oh	76	2003	Netherlands	Sociology	To examine why older adults living in urban neighborhoods consider moving.	Quantitative study, case series	1123 (aged≥ 65)	H
Oswald *et al*.^†^	28	2007	Germany-Hungary-Leetonia-Sweden-UK	Psychology (3), medicine (2), occupational therapy (2), gerontology, rehabilitation, sociology	To examine and compare the relationship between objective and perceived housing and aspects of healthy ageing among older adults from four countries.	Quantitative study, incidence/prevalence study	1918 (aged 75–89, mean age 83)	M
Painter and Lee	77	2009	USA	Urban planner, design	To identify the reasons why older households make housing transitions.	Quantitative study, case series, longitudinal	4018 (aged≥ 50)	H
Perks and Haan	78	2010	Canada	Kinesiology, sociology	To analyze how social support networks, health and economic characteristics have shaped the residential choices of older adults, and predict how they are likely to do so in the future.	Quantitative study, cohort projection	15 755 (aged 55–75)	H
Reed *et al*.	79	1998	UK	Nursing (2), anthropology	To explore the process of moving into nursing and residential homes for older adults.	Qualitative method, phenomenology, longitudinal	46 (from older adult’s designed housing)	M
Renault^§^	29	2007	France	Administration	To examine the impact of illness and handicap on people’s lives and activities (translated from French).	Quantitative study, incidence/prevalence study, longitudinal	11118 (aged≥ 65)	H
Sabia	80	2008	USA	Economy	To estimate the effects of family composition changes, health conditions, housing characteristics, and local policies and amenities on aging-in-place decisions by older homeowners.	Quantitative study, incidence/prevalence study, longitudinal	12 061 (aged 50–89)	H
Sergeant *et al*.	81	2008	USA	Specialised education, sociology	To examine the relationship between older adults’ expectations to move and actual residential relocation in the community or to a nursing facility within two years.	Qualitative study, case study	30 (aged≥ 60)	H
Serow *et al*.	82	1996	USA-Germany	Demography, geography, sociology	To identify some of the principal differences and similarities in the migration and spatial redistribution behavior of older adults between two countries.	Quantitative method, case series	1048 (aged ≥ 55)	M
Sheehan and Karasik	83	1995	USA	Human development	To examine factors related to older adults’ decisions to move to a continuing care retirement community.	Quantitative method, Incidence/prevalence	184 CCRC residents (mean age, 79.7) 246 CCRC waiting list (mean age 73.4)	H
Shen and Perry	84	2014	USA	Social work (2)	To evaluate the relationship between volunteering in a community and relocation.	Quantitative study, incidence/prevalence study	9220 (aged≥ 50, mean age 74)	H
Smith and Sylvestre	85	2008	Canada	Social work (2)	To determine the effects of neighborhood and individual change on the personal outcomes of recent older movers to government-subsidized senior citizen apartment buildings.	Quantitative study, case report, longitudinal	137 (aged 55–89)	H
Somenahalli and Shipton	86	2013	Australia	Engineering, geology	To examine the distribution of older adults and accessibility to essential services.	Quantitative study, case series	ND Comparison between 4 age groups (55–64, 65–74, 75–84, 85+)	H
Sommers and Rowell	87	1992	USA	Politic science, sociology	To identify factors which differentiate elderly residential movers from nonmovers.	Quantitative method, cohort study, longitudinal	2950 (aged ≥ 70, mean age 77)	M
Stoeckel and Porell	88	2010	USA	Psychology, health economy	To investigate the relationship between falls and expected probability of housing relocation among older adults.	Quantitative study, incidence/prevalence study	8462 (aged≥ 65, mean age 74)	H
Sweaney *et al*.	89	2004	USA	Administration, economy (2)	To examine perceived changes in housing quality and the neighborhood characteristics in order to further the understanding of the housing conditions that today’s older adults face.	Quantitative study, incidence/prevalence study	780 (aged ≥ 55)	H
Sylvestre*	106	2005	Canada	Social work	To investigate the effect of changes in local environmental settings, individual attributes, and local travel behavior on the personal outcomes of older movers to government-subsidized senior housing projects.	Quantitative study, case series, longitudinal	149 (aged≥ 65)	M
Tang and Pickard	90	2008	USA	Social work (2)	To examine associations between the perceived awareness of community-based long-term care and supportive services and the anticipation of aging in place and relocation.	Quantitative study, incidence/prevalence study	4611 (aged ≥50)	M
Tanner *et al*.	91	2008	Australia	Occupational therapy (2), social work	To examine the home modification experience of older adults living in the community who are recipients of this service.	Qualitative study, phenomenology	12 (aged≥ 65)	H
Tenamoc*	107	2000	USA	Sociology	To look at the role assisted living plays in continuum of care services for older frail adults and explore their process of making a decision to move from their traditional living environments to one of assisted living.	Qualitative study, qualitative description, longitudinal	22 older adults (mean age 86) 21 family members 22 assisted living administrators	H
Tyvimaa and Kemp	92	2011	Finland	Engineering, sociology	To explore the factors influencing residential decisions of Finnish seniors.	Qualitative study, case study	37 (aged ≥ 55)	M
VanderHart	93	1993	USA	Economy	To determine what factors are most important in the home equity decisions of older homeowners.	Quantitative method, Incidence/prevalence, longitudinal	6400 (aged ≥ 50, mean 68.5)	M
VanderHart^§^	110	1995	USA	Economy	To shed on light on the housing changes and the most important considerations in older adults’ housing decisions.	Quantitative method, Incidence/prevalence, longitudinal	1400 (aged ≥ 50)	M
VanderHart	94	1998	USA	Economy	Provide a dynamic empirical investigation of the housing decisions of older households.		12 323 (aged ≥ 50)	M
VanderHart	95	2002	USA	Economy	To determine the importance of financial, demographic, and housing market factors to older migrants’ choice among several tenure alternatives.	Quantitative study, Incidence/prevalence, longitudinal	195 (aged ≥ 55, mean age 67.7)	M
Walker and McNamara	96	2013	Australia	Occupational therapy (2)	To identify issues healthy older adults face when relocating to retirement living, what strategies they used during this process, how they maintained a sense of home, and the potential for occupational therapy involvement.	Qualitative study, grounded theory	16 (aged≥ 65)	H
Walters	97	2002	USA	Demography	To evaluate the impact of origin and destination housing characteristics on the internal migration of retirees.	Quantitative study, case series	732 (aged≥ 65)	H
Weeks *et al*.	98	2012	Canada	Gerontology, psychology, sociology+anthropology	To evaluate how contextual, push and pull factors influence preferences of older adults to relocate.	Quantitative study, incidence/prevalence study	1015 (aged≥ 65)	M
Yun*	108	2003	USA	Economy	To evaluate the effect of economic, demographic and health factors on several housing decisions.	Quantitative study, case report	1485 (aged≥ 75)	H
Zimmer and Korinek	99	2008	Cambodia-China- Philippines- Singapore- Taiwan-Thailand	Sociology (2)	To evaluate the probability that older adults live in the same household or nearby an adult child and how this probability fluctuates by the number of children, rural/urban residence, and several other covariates.	Quantitative study, incidence/prevalence study	24867 (aged≥ 65)	H

*Doctoral thesis reviewed by peer review committee.

^§^ Book chapter published in a book peer-reviewed by editorial committees.

^†^ Papers describing the results of the Enable-Age Project.

^††^ Papers describing the results of the Pathway to Life project.

^‡^ Papers describing the longitudinal study in Lord’s doctoral thesis.

Two hundred and ten (210) distinct authors signed or co-signed the 86 studies, among whom 43% (n = 90) were in social sciences [mostly psychology (n = 29), sociology (n = 19) and social work (n = 15)]; 29% (n = 60) in health sciences [two thirds in occupational therapy (n = 20), nursing (n = 10) and medicine (n = 9)]; 13% (n = 27) in economy and administration [dominated by economics, n = 21]; 5% (n = 11) in planning, architecture or design [about half in architecture (n = 6)], and 10% (n = 22) in other research domains [dominated by engineering (n = 6) and geography (n = 5)]. Most common disciplinary affiliations were thus with psychology, occupational therapy and economics. Two-thirds of the studies (n = 57) were either signed by one author (n = 30) [29, 40, 46, 49, 59, 62–65, 68–70, 72, 74, 76, 80, 93–95, 97, 100–108, 110], by co-authors in the same discipline (n = 18) [10, 41–45, 52, 54, 55, 67, 73, 75, 83–85, 90, 96, 99], or by co-authors in the same research domains (n = 9) [33, 47, 48, 51, 57, 58, 77, 87, 98]. Among the remaining 29 studies (34%), 21 were co-signed by authors from two different research domains [21–25, 28, 30, 34, 36, 39, 50, 53, 56, 61, 71, 78, 79, 81, 82, 86, 88, 89, 91, 92, 109] (nine from social or health sciences) and eight by authors from three or more research domains [26, 27, 31, 32, 35, 37, 38, 60, 66].

Nine studies involved researchers in planning, architecture or design [26, 27, 35, 66, 77, 100–102, 104, 109], of which four were in collaboration with at least one other research domains [26, 27, 35, 66, 109].

A total of 60% of the studies were quantitative [10, 21, 24, 29, 30, 32, 36–38, 40–45, 50, 52, 53, 55, 56, 59, 60, 63, 64, 66–69, 73, 75–78, 80, 82–90, 93–95, 97–99, 104, 106, 108, 110], mostly descriptive. On the other hand, 30% of the studies were qualitative [31, 33, 34, 39, 46, 48, 49, 51, 57, 58, 62, 65, 70–72, 74, 79, 81, 91, 92, 96, 102, 103, 105, 107, 109]. Eight studies used mixed methods [22, 23, 25–28, 35, 47, 54, 61, 100, 101].

More than half of the studies (n = 47) looked at the housing decisions of older adults only after they had made a choice [10, 26, 27, 31–35, 37, 39, 41–49, 56, 58, 59, 62, 63, 65–67, 70, 71, 73–75, 78, 80, 82, 85–87, 91, 92, 96, 99, 102–104, 107–109]. Four more examined only during the decision process [51, 60, 64, 69]. Three out of ten studies considered different steps in the housing decision process, whether before, during or after the decision was made [21–25, 28–30, 36, 40, 50, 53–55, 57, 61, 77, 79, 81, 83, 84, 89, 93–95, 97, 100, 105, 106, 110]. Twelve studies addressed the housing decision as a purely hypothetical choice, and did not record whether or when the older adults had made an actual decision [38, 52, 60, 68, 72, 76, 88, 90, 93, 98, 101, 105]. Among the studies of non-hypothetical housing decisions, 38 looked at them from a post-relocation perspective [31–33, 41–44, 46–51, 56, 58, 59, 62, 63, 65, 70, 71, 73, 74, 79, 81–83, 85, 89, 92, 96, 97, 102–104, 106–108], with 6 of them not specifying the type of destination (e.g. private dwelling, cooperative, assisted living, nursing home) [41–43, 56, 73, 89]. In an additional 31 studies, the samples of older adults had either chosen to relocate or stay at home [10, 21–30, 34–37, 39, 40, 45, 53–55, 57, 61, 64, 67, 69, 75, 77, 78, 84, 86, 87, 94, 95, 99, 110]. Five studies looked at the housing decision only among those who had decided to stay at home [66, 80, 91, 100, 109].

In about two-thirds of the studies (n = 57), most factors influencing the housing decision were reported by study participants or by researchers through interviews, census questionnaires or observational grids [10, 30, 31, 34–36, 38, 39, 42–44, 46, 48–58, 60, 62, 63, 65, 66, 69–72, 74, 78–81, 86–96, 98–100, 102, 103, 105, 107, 109, 110]. Sixteen other studies referred to factors being objectively measured [29, 37, 40, 41, 45, 59, 64, 67, 68, 73, 75–77, 82, 85, 97] and 13 combined both self-reported and objectively measured data [21–28, 32, 33, 47, 61, 83, 84, 101, 104, 106, 108]. Overall, nine studies specified the use of at least one validated measurement instrument or scale for data collection [21–28, 30, 44, 50, 61, 86, 101]. These scales assessed either the physical or mental health of older adults and their caregivers, as well as aspects of their dwellings.

Sample sizes ranged from 91 to 502 075 participants in quantitative studies, from eight to 120 in qualitative studies, and from 10 to 8022 in mixed-methods studies. In two studies using data from national surveys, sample sizes were not recorded [43, 86]. Eight studies specified additional samples of family members, friends, health professionals, needs assessors or service coordinators [31, 38, 39, 57, 60, 61, 65, 107].

### The characteristics of participants and their housing

In almost half of the studies (n = 41), the minimum age of participants was under 65 years old [21, 24, 26, 27, 30, 32, 34, 35, 41, 42, 44, 45, 53, 54, 56, 59, 67–70, 72–74, 77, 78, 80–82, 84–86, 89, 90, 92–95, 101, 104–106, 109, 110]; almost the same proportion (n = 38) excluded people under 65 years old [10, 29, 31, 33, 36, 37, 39, 40, 43, 46, 48, 50–52, 55, 57, 58, 61, 63–66, 71, 75, 76, 83, 87, 88, 91, 96–100, 102, 103, 107, 108] and two excluded people under 75 years old (n = 2) [22, 23, 25, 28, 62]. Four studies only specified their sample mean or median age which was over 65 years old [38, 47, 49, 60]. In one case, the sample was composed exclusively of residents living in housing designed for older adults with or without additional care [79]. Among all studies, 10 targeted populations with a large age range with analyses per age group [26, 27, 29, 35, 41, 44, 53, 56, 69, 80, 86].

Less than 40% assessed the autonomy levels of participants (n = 34). They were either frail (n = 17) [22, 23, 25, 28, 29, 31, 32, 39, 48, 49, 54, 55, 61, 62, 66, 70, 88, 91, 107, 109], in relatively good health (n = 6) [33, 36, 64, 71, 98, 103], in very good health (n = 2) [21, 24, 96] or showed varying levels of health (n = 9) [26, 27, 35, 38, 60, 80, 83, 90, 100, 108].

Almost three-quarters of the studies (n = 63) did not specify the residential sector type (urban, suburban or rural) in which participants lived [10, 29, 30, 32, 33, 36, 37, 39–41, 44–55, 57–60, 62–64, 67–71, 73–77, 79–81, 83, 84, 87–91, 93–98, 100, 103–108, 110]. Five studies reported on participants living in suburban areas [26, 27, 38, 86, 101, 109], two in urban areas [22, 23, 25, 28, 61] and 16 included two or more residential environments [21, 24, 31, 34, 35, 42, 43, 56, 65, 66, 72, 78, 82, 85, 92, 99, 102]. Over half of the studies investigated people living in traditional housing (i.e. not specifically designed for older adults) at the beginning of the study (n = 49) [10, 21–28, 30, 35, 36, 38, 41–45, 51, 53, 55, 60, 61, 64, 66, 68, 69, 71–77, 80–82, 84, 87–90, 93–95, 97–101, 104, 105, 109, 110]; 24 exclusively targeted housing designed for older adults, with or without additional care [31–33, 37, 39, 46–50, 58, 62, 63, 65, 70, 83, 85, 91, 92, 96, 102, 103, 106, 107], 11 others targeted both [29, 34, 40, 52, 54, 56, 57, 59, 78, 79, 108] and two studies did not specify [67, 86]. Almost one-third of studies (n = 27) did not record the specific type of housing participants were living in (detached house, condominium or apartment, assisted living, congregate housing, etc.) [35, 41–44, 53, 55, 60, 67–69, 72, 76, 80–82, 84, 86, 88–90, 93–95, 97, 98, 110].

Over three-quarters of the studies specified the tenure status of their participants. In 10 studies, participants were all homeowners [26, 27, 35, 38, 45, 64, 73, 77, 80, 101, 109] and in 18 others, private renters [31–33, 43, 46–49, 51, 62, 65, 70, 79, 83, 102–105]. Seven studies considered other residential arrangements such as subsidized housing or long-term care facilities [37, 40, 58, 85, 91, 99, 106]. The remaining studies included participants of any tenure status (n = 33) [10, 21–25, 28–30, 34, 36, 39, 42, 50, 53, 54, 56, 57, 59, 69, 71, 74, 78, 87–90, 92–96, 98, 100, 107, 108, 110], of which 21 studies included renters and owners specifically [10, 21–25, 28, 29, 30, 34, 42, 50, 53, 59, 69, 71, 74, 78, 87–90, 92, 96, 98]. However, only four of them compared the factors influencing housing decisions between those two tenure types [23, 30, 94, 95]. Tenure status was not reported in 18 studies [41, 44, 52, 55, 60, 61, 63, 66–68, 72, 75, 76, 81, 82, 84, 86, 97].

Seven studies reported on the average time older adults had lived in their current dwelling [26, 27, 52, 65, 67, 71, 83, 107]. Three specified the main transportation mode used by participants in their daily life [21, 24, 26, 27, 72].

### The quality of the studies

Following the quality appraisal of all studies, 59 scored as of high quality [10, 29–32, 34, 36, 37, 39–41, 43, 46–48, 51, 53–60, 62–66, 68, 70, 73–78, 80, 81, 83–86, 88, 89, 91, 93–97, 99, 100, 102, 103, 105, 107, 108, 110] and 24 of medium quality [33, 35, 38, 42, 44, 45, 49, 50, 52, 61, 67, 69, 71, 72, 79, 82, 87, 90, 92, 98, 101, 104, 106, 109]. Two mixed-method studies had different quality rating for their quantitative versus qualitative parts [22, 23, 25–28], and two quantitative sub-studies within a single research project had different quality evaluations [21, 24]. No low-quality studies were identified. All 86 retrieved studies were thus considered for analysis.

### Synthesis of results

#### Factors influencing the housing decision

A total of 88 potential factors of influence on older adults’ housing decisions were extracted from the 86 studies. Of these 88 potential factors, 78% were individually addressed in less than one quarter of the studies and 42% in less than one out of ten. Our previous study [111] reported on a total of 55 influential factors linked to the meaning and experience of home. By adding a dimension to the initial model associated with the socioeconomic and health-related factors, as well as refining the extraction, this paper brings the total number of factors up to 88. The effect of each of these factors of influence on the housing decision was also assessed. Table 3 reports the number of studies reporting on each of the 88 factors found to influence the housing decision of older adults, push and pull factors combined. The reported effect of each factor is recorded globally but also according to the study design (quantitative, qualitative, mixed).

**Table 3:** Factors associated with the housing decision, classified according to the dimensions of the meaning and experience of home, by type of research design, strength of evidence and effect (N = 86).

Factors classified according to six dimensions of the experience and meaning of home	Quantitative method n = 52*		Mixed method n = 8		Qualitative method n = 26		Total number of studies N = 86					Publication citations
	Effect	No effect identified	Effect	No effect identified	Effect	No effect identified	Effect		No effect identified		Total	
	n	n	n	n	n	n	n	%	n	%	N	
Feeling of control over decision and environment	5	0	4	0	12	0	21	100%	0	-	21	[22, 23, 28, 29, 31, 33, 39, 47, 51, 54, 58, 62–64, 67, 70, 81, 89, 91, 96, 100, 102, 103]
Relation to neighbors	7	0	2	0	6	0	15	100%	0	-	15	[26, 34, 35, 38, 44, 53, 69, 70, 74, 76, 80, 88, 91, 102]
Personal identity	0	0	1	0	12	0	13	100%	0	-	13	[31, 46, 48, 58, 62, 70, 74, 91, 96, 100, 102, 103, 105]
Routine and habits	2	0	3	0	6	0	11	100%	0	-	11	[22, 25, 26, 31, 46, 58, 63, 85, 100, 102, 103, 105]
Familiarity with place	0	0	3	0	6	0	9	100%	0	-	9	[22, 25, 26, 33, 70, 79, 91, 100, 102, 103]
Housing market	6	0	0	0	2	0	8	100%	0	-	8	[30, 69, 71, 75, 82, 96, 97, 108]
Convenient dwelling	1	0	3	0	3	0	7	100%	0	-	7	[22, 23, 28, 32, 35, 91, 100, 102, 109]
Doctor and health professional opinion	2	0	1	0	3	0	6	100%	0	-	6	[31, 38, 39, 60, 61, 107]
Feeling of comfort	1	0	3	0	2	0	6	100%	0	-	6	[22, 28, 35, 100, 102, 103, 106]
Investment return	3	0	0	0	2	0	5	100%	0	-	5	[41, 45, 71, 105, 108]
Maintenance requirements	7	1	5	0	13	0	25	96%	1	4%	26	[21, 24, 26, 31–33, 35, 44, 47, 49, 51, 53, 57, 64, 65, 71, 74, 80, 81, 83, 91, 92, 96, 98, 100, 101, 105]
Feeling of independence	4	1	3	0	16	0	23	96%	1	4%	24	[21, 23–25, 28, 31, 33, 34, 39, 44, 47, 49, 51, 52, 57, 58, 62, 67, 71, 72, 83, 91, 96, 100, 102, 103, 105]
Social activities	6	1	2	0	9	0	17	94%	1	6%	18	[22, 25, 31, 32, 34, 44, 46, 47, 68, 74, 76, 83, 84, 92, 96, 102, 103, 105, 106]
Proximity of services	6	1	3	0	5	0	14	93%	1	7%	15	[26, 27, 31, 32, 35, 41, 48, 69, 79, 85, 86, 89, 92, 100, 105, 106]
Domestic activities (including IADL)	7	2	4	0	9	0	20	91%	2	9%	22	[22, 23, 27–29, 31, 32, 39, 48, 54, 55, 57, 61, 62, 67, 68, 81, 84, 85, 88, 91, 96, 97, 102]
Dwelling potential adaptability	3	1	3	0	4	0	10	91%	1	9%	11	[29, 32, 35, 54, 71, 81, 90, 91, 98, 100, 109]
Tenure status	19	3	2	0	5	1	26	90%	3	10%	30	[10, 23, 29–33, 37, 42, 45, 49, 50, 53, 56, 59, 64, 74, 77, 84, 87, 89, 90, 93, 97, 98, 100, 104, 105, 107, 110]
Adapted dwelling	7	0	4	1	8	1	19	90%	2	10%	21	[21, 23, 26, 28, 30, 32, 33, 35, 38, 39, 53, 61, 70, 71, 74, 84, 91, 92, 96, 100, 106, 109]
Social and support network	8	2	3	0	8	0	19	90%	2	10%	21	[26, 28, 31, 34, 38, 44, 57, 67–69, 73, 84, 92, 100, 102–108]
Proximity of siblings	5	1	1	0	3	0	9	90%	1	10%	10	[29, 32, 39, 54, 67, 74, 78, 81, 84, 85]
Trigger event	2	1	1	0	6	0	9	90%	1	10%	10	[31, 39, 45, 49, 54, 57, 73, 83, 96, 105]
*Pressure from family*^!^	*2*	*2*	*5*	*0*	*9*	*0*	*16*	*89%*	*2*	*11%*	*18*	[25, 26, 29, 32, 33, 39, 47, 48, 54, 60–62, 65, 71, 81, 104, 105, 107]
Housing costs	11	2	3	0	2	0	16	89%	2	11%	18	[26, 30, 32, 35, 38, 43, 45, 71, 75, 80, 82, 86, 89, 93, 100, 102, 108, 110]
Geographic location	19	5	4	0	13	0	36	88%	5	12%	41	[10, 24, 26, 27, 30–33, 35, 41–44, 46, 51–54, 56, 57, 66, 70, 72, 76–82, 84, 85, 89, 92, 96–100, 102, 105, 106]
Proximity and presence of friends	9	3	5	1	13	0	27	87%	4	13%	31	[25, 26, 31, 33–35, 38, 44, 46–48, 51, 57, 67, 71, 74, 76, 77, 82, 84, 85, 88, 89, 96, 100–106]
Programs and services	5	2	4	0	5	0	13	87%	2	13%	15	[25, 31, 44, 49, 54, 55, 60, 61, 73, 83, 87, 91, 100, 105, 108]
Proximity and presence of children	16	6	6	0	16	0	38	86%	6	14%	44	[21, 24, 25, 29–35, 40, 44, 48, 49, 51, 54, 57–59, 61, 65, 67–69, 71, 74, 77, 80–82, 84, 85, 87–89, 93, 96, 99–106]
Feeling of security/fear	5	4	4	0	15	0	24	86%	4	14%	28	[22, 25, 26, 32–35, 44, 48, 51, 58, 59, 62, 63, 70, 72, 83–85, 91, 92, 96, 97, 100, 102–105, 107]
Expression of family roles	10	3	3	0	5	0	18	86%	3	14%	21	[21, 25, 30, 31, 33, 40, 44, 67, 68, 71, 81, 84, 85, 87, 88, 91, 99–101, 104, 106]
*Values and Religion*^!^	*1*	*1*	*1*	*0*	*4*	*0*	*6*	*86%*	*1*	*14%*	*7*	[54, 59, 68, 81, 96, 103, 105]
Personal care activities (including ADL)	9	3	3	1	9	0	21	84%	4	16%	25	[23, 24, 26–33, 39, 48, 54, 55, 61, 72, 74, 75, 81, 83–86, 88, 91, 96, 98]
Satisfaction	6	3	3	0	6	0	15	83%	3	17%	18	[21, 23, 28, 33, 54, 59, 62, 63, 70, 76, 82, 85, 88, 91, 98, 101, 102, 105, 106]
Neighborhood beauty and general quality	2	1	2	0	1	0	5	83%	1	17%	6	[34, 35, 67, 89, 90, 101]
Knowledge of housing options	1	0	0	1	4	0	5	83%	1	17%	6	[39, 51, 61, 90, 96, 107]
*Needs anticipation*^!^	*3*	*0*	*1*	*1*	*1*	*0*	*5*	*83%*	*1*	*17%*	*6*	[21, 23, 58, 60, 61, 83]
*Attachment/sense of belonging to dwelling*^!^	*2*	*3*	*2*	*1*	*14*	*0*	*18*	*82%*	*4*	*18%*	*22*	[22, 23, 25, 28, 34, 44, 46–48, 51, 52, 58, 70–74, 76, 88, 91, 96, 100, 102, 103, 105]
*Feeling of intimacy*^!^	*0*	*2*	*2*	*0*	*7*	*0*	*9*	*82%*	*2*	*17%*	*11*	[21, 22, 34, 44, 46, 47, 58, 74, 102, 103, 105]
General health status	16	8	6	0	10	0	32	80%	8	20%	40	[21, 24, 25, 28, 30–32, 35, 37, 38, 47, 48, 50, 51, 53, 55, 58–61, 64–67, 77, 78, 82, 83, 85, 87, 88, 90–92, 98, 100, 101, 103–105, 107, 108]
*Timing*^!^	*2*	*3*	*0*	*0*	*10*	*0*	*12*	*80%*	*3*	*20%*	*15*	[31, 32, 39, 45, 46, 49, 51, 60, 63, 68, 70, 71, 81, 96, 105]
Coping strategies	0	1	4	1	4	0	8	80%	2	20%	10	[22, 26, 27, 46, 54, 61, 63, 72, 96, 100, 103]
Housing value	7	2	0	0	1	0	8	80%	2	20%	10	[41, 45, 75, 77, 82, 93, 95, 97, 105, 108]
Past residential experiences	4	2	2	0	2	0	8	80%	2	20%	10	[22, 25, 32, 47, 48, 56, 77, 80, 88, 102, 104]
*Availability of the family*^!^	*1*	*1*	*2*	*0*	*1*	*0*	*4*	80%	*1*	20%	*5*	[26, 61, 63, 81, 83]
*Neighborhood accessibility*^!^	*2*	*0*	*1*	*1*	*1*	*0*	*4*	80%	*1*	20%	*5*	[27, 79, 100, 104, 106]
Housing building type	8	6	4	0	9	0	21	78%	6	22%	27	[23, 24, 30, 31, 36, 37, 39, 43, 46, 49, 50, 54, 59, 69, 74, 84, 86, 92, 93, 97, 98, 100–102, 104, 105, 107]
*Expression of social role*^!^	*3*	*1*	*1*	*1*	*3*	*0*	*7*	*78%*	*2*	*22%*	*9*	[26, 38, 40, 60, 71, 84, 96, 100, 102]
Dwelling size	8	6	5	0	6	0	19	77%	6	23%	25	[21–24, 26, 30, 32, 35, 44, 53, 54, 59, 64, 65, 70, 71, 75, 77, 79–82, 86, 92, 93, 100, 104]
Household composition	25	11	2	0	7	0	34	76%	11	24%	45	[10, 21, 24, 25, 29–32, 36, 37, 40, 43, 46, 48–50, 57, 59, 64, 68, 73, 75, 77, 78, 80–85, 87–90, 92–95, 97–100, 104, 106, 108, 110]
Physical limitations	20	15	5	0	19	0	44	75%	15	25%	59	[21, 23, 24, 28–33, 36–41, 47–49, 51, 54, 55, 57–62, 64, 66–69, 71, 75–78, 80–84, 87, 88, 91–100, 102, 103, 105, 107–110]
Access to public transport	3	2	1	0	2	0	6	75%	2	25%	8	[44, 72, 80, 89, 90, 92, 100, 106]
Equity	5	2	0	0	1	0	6	75%	2	25%	8	[45, 80, 93–95, 97, 107, 110]
Relocation associated costs	4	1	0	1	2	0	6	75%	2	25%	8	[25, 30, 32, 44, 74, 94, 97, 105]
*Presence of public facilities*^!^	*1*	*1*	*0*	*0*	*2*	*0*	*3*	*75%*	*1*	*25%*	*4*	[24, 39, 70, 89]
*Neighborhood status*^!^	*5*	*4*	*1*	*1*	*8*	*0*	*14*	*74%*	*5*	*26%*	*19*	[21, 24, 30, 32, 34, 44, 46, 49, 51, 70, 74, 76, 80, 84, 86, 92, 97, 100, 101, 105]
*Ethnic background*^!^	*11*	*3*	*1*	*0*	*0*	*2*	*12*	*71%*	*5*	*29%*	*17*	[10, 31, 37, 40–42, 68, 75, 76, 78, 80, 84, 89, 99, 100, 103, 108]
No. of years in current dwelling/neighborhood	7	5	3	0	2	0	12	71%	5	29%	17	[21–23, 25, 30, 32, 35, 45, 53, 59, 69, 76, 87, 88, 93, 98, 100, 105, 107]
Active economic assets	3	2	0	0	2	0	5	71%	2	29%	7	[92–95, 97, 107, 110]
*Residential preconceptions*^!^	*1*	*2*	*0*	*0*	*4*	*0*	*5*	*71%*	*2*	*29%*	*7*	[46, 49, 59, 63, 88, 103, 105]
Current/anticipated income	23	13	4	1	6	1	33	69%	15	31%	48	[10, 21, 24, 25, 30, 31, 33, 35, 37, 38, 40–43, 45, 47, 49, 53, 56, 59, 66, 73–78, 80, 81, 84–90, 93–98, 100, 101, 104–106, 108, 110]
Residential aspirations	5	3	3	0	2	0	9	69%	3	31%	13	[21, 32, 35, 45, 51, 61, 63, 67, 68, 73, 82, 100, 105]
Functional mixity	4	3	0	0	2	0	6	67%	3	33%	9	[24, 32, 44, 70, 86, 89, 96, 97, 106]
*Informal help available*^!^	*3*	*3*	*1*	*0*	*2*	*0*	*6*	*67%*	*3*	*33%*	*9*	[26, 40, 51, 57, 60, 63, 66, 83, 108]
*Dwelling beauty and general condition*^!^	*2*	*2*	*1*	*0*	*1*	*0*	*4*	*67%*	*2*	*33%*	*6*	*[*58, 60, 61, 80, 82, 89*]*
*Feeling stressed*^!^	*1*	*2*	*0*	*0*	*3*	*0*	*4*	*67%*	*2*	*33%*	*6*	*[*46, 51, 59, 63, 67, 103*]*
Residential density	3	2	1	0	0	0	4	67%	2	33%	6	*[*41, 77, 86, 97, 99, 100*]*
*Residential preferences*^!^	*4*	*4*	*0*	*0*	*3*	*0*	*7*	*63%*	*4*	*36%*	*11*	[21, 44, 49, 59, 63, 67, 68, 82, 88, 105, 107]
Age	26	17	4	0	3	2	33	63%	19	37%	52	[10, 21, 23, 27, 29–32, 35–38, 40–42, 45, 50, 53, 59, 60, 64, 66, 68, 69, 73, 75–78, 80–84, 86–89, 91–100, 102, 104, 106, 108, 110]
Number of children	5	3	0	0	0	0	5	63%	3	37%	8	[30, 40, 41, 75, 87, 93, 99, 108]
*Presence of garden/yard*^!^	*0*	*2*	*2*	*1*	*3*	*0*	*5*	*63%*	*3*	*37%*	*8*	[26, 35, 44, 48, 89, 100, 102, 105]
Housing taxes	4	2	0	0	0	0	4	63%	2	33%	6	[30, 41, 45, 80, 93, 95]
*Number of storeys*^!^	*1*	*3*	*1*	*1*	*4*	*0*	*6*	*60%*	*4*	*40%*	*10*	[44, 51, 54, 71, 74, 82, 86, 92, 100, 104]
Education	13	10	0	1	1	0	14	56%	11	44%	25	[10, 21, 24, 31, 40, 41, 55, 59, 67, 68, 75, 76, 78, 80, 84, 85, 87–90, 93, 95, 99, 100, 104, 108]
Employment /prior occupation	*6*	*7*	*1*	*0*	*1*	*1*	*8*	*50%*	*8*	*50%*	*16*	[30, 35, 40, 53, 67, 76, 78, 80, 89, 92–94, 97, 99, 102, 110]
Traffic and car facilities	1	2	1	0	0	0	2	50%	2	50%	4	[80, 89, 100, 106]
Gender	*12*	*21*	*1*	*1*	*1*	*2*	*14*	*37%*	*24*	*63%*	*38*	[10, 21, 23, 30–32, 36, 37, 40, 41, 45, 49, 53, 55, 59, 60, 66, 69, 73, 75, 76, 78, 80, 83–85, 88–90, 93, 95, 98–100, 102, 106, 108, 110]
Mortgage/reverse mortgage	1	2	0	0	0	0	1	33%	2	67%	3	[30, 93, 95]
Adaptation costs	1	0	1	0	2	0	4		0		4	[29, 71, 81, 100]
Caregivers characteristics	2	0	1	0	1	0	4		0		4	[40, 57, 61, 66]
Climate conditions	1	0	2	0	1	0	4		0		4	[41, 100, 101, 107]
Friends/sibling experience	0	0	1	0	3	0	4		0		4	[46, 51, 101, 105]
Housing offers	1	1	1	0	1	0	3		1		4	[32, 95, 101, 107]
Experience of falls	1	0	1	0	1	0	3		0		3	[61, 88, 107]
Location in the building	1	0	1	0	1	0	3		0		3	[24, 54, 70]
Presence or absence of caregivers	0	0	0	0	2	0	2		0		2	[57, 107]
Presence of green spaces	2	0	0	0	0	0	2		0		2	[44, 97]
Social pressure	0	0	0	0	2	0	2		0		2	[71, 81]
Housing and care services costs	0	0	0	0	1	0	1		0		1	[65]
Having a pet	1	0	0	0	0	0	1		0		1	[44]

Number of independent studies addressing each factor are not mutually exclusive.

*n = number of independent studies.

^!^ Factors for which a discrepancy was identified between studies with different methods.

Among all 88 potential factors of influence investigated, having a *mortgage or reverse mortgage* was found to have an effect on older adults’ housing decisions in 33% of the assessed studies, *gender* in 37% of them, *education*, *employment* and *traffic and car facilities* in about half of them. For 12 additional factors, evidence was insufficient to discuss any trend since they were addressed in fewer than three quantitative studies or fewer than five studies of any design method. The remaining 71 factors were found to have an overall effect on older adults’ housing decisions in at least 60% of the studies in which they were considered, although 19 of them show discrepancies between quantitative, qualitative and mixed methods study designs.

Of the 71 factors found to have an overall effect on the housing decision, 21 of them had an overall level of agreement among studies ranging from 90% to 100%. Thirty-two additional factors were found to influence older adults’ housing decisions, with levels of agreement of 75% to 89% between studies, and the remaining 18 factors identified as having an effect on the housing choice had degrees of agreement ranging from 60% to 74%.

#### The dimensions of the experience and meaning of home influencing the housing decision

**Fig 2:**
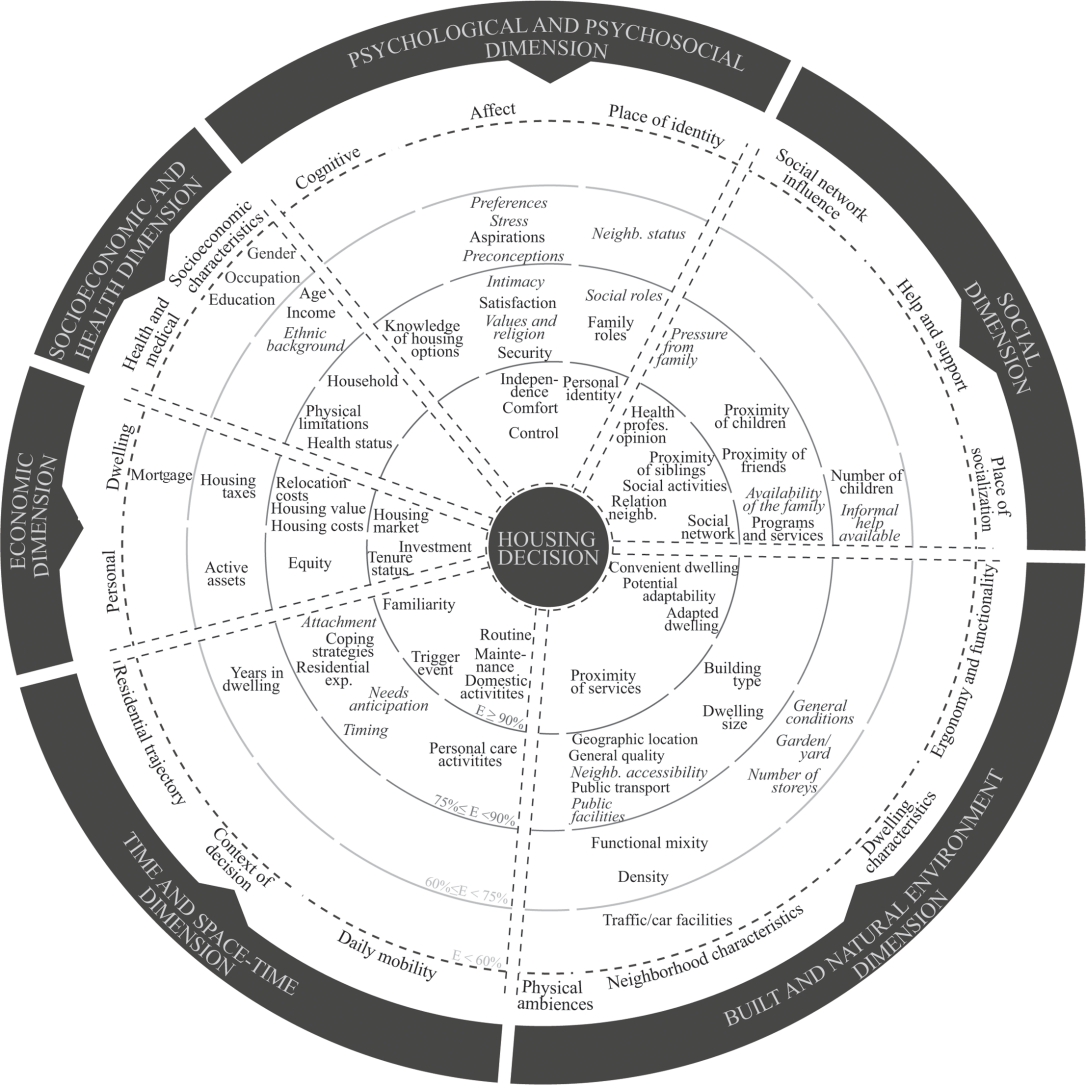
Factors influencing the housing decision of older adults, classified by the meaning and experience of home dimensions. (A) Factors are classified by their overall reported effect (E) on the housing decision of older adults. The ones closer to the center have a greater effect. (B) Italics: Factors for which a discrepancy was identified between studies with different methods.

The 88 factors were then classified within the six dimensions of the experience and meaning of home (Fig 2). Several potential factors of influence associated with the socioeconomic- and health-related dimensions of home were considered simultaneously in most studies, while just a few of the factors associated with the other five dimensions were explored per study. Indeed, a total of 79 studies considered factors related to the socioeconomic- and health-related dimension [10, 21, 23–25, 27–33, 35–43, 45–51, 53–62, 64–69, 71, 73–78, 80–110], 71 to the built and natural environment dimension [10, 21–24, 26–39, 41–44, 46, 48–54, 56–61, 64–67, 69–72, 74–82, 84–86, 89–93, 95–102, 104–107, 109], 66 to the social dimension [21, 22, 24–26, 28–35, 38–41, 44, 46–49, 51, 53–55, 57–63, 65–71, 73–78, 80–85, 87–89, 91–93, 96, 99–108], 65 to the time and space-time related dimension [21–35, 39, 44–49, 51–65, 67–77, 79–81, 83–88, 91–93, 96–98, 100–105, 107], 63 to the psychological and psychosocial dimension [21–26, 28–35, 38–40, 44–49, 51, 52, 54, 57–64, 67, 68, 70–74, 76, 80–92, 96–107], and 51 to the economic dimension [10, 23–26, 29–33, 35, 37, 38, 41–45, 49, 50, 53, 56, 59, 64, 65, 69, 71, 74, 75, 77, 80–82, 84, 86, 87, 89, 90, 92–98, 100, 102, 104, 105, 107, 108, 110]. Factors belonging to four of the six dimensions of the experience and meaning of home (if the economic and the socioeconomic/health dimensions are excluded) were seldom considered in quantitative studies, except for a few individual factors. Conversely, factors belonging to the economic and socioeconomic/health dimensions were mostly explored using quantitative designs. Qualitative and mixed-methods studies typically considered a more diverse range of potential factors of influence on housing decisions.

Discarding the 19 factors with discrepancies between study methods, 52 factors of influence identified remain as having an effect on the older adults’ housing decision in at least 60% of the studies assessing them. Those factors cover all six dimensions of the meaning and experience of home. Eleven were related to the built or natural environment of the dwelling, nine were of a psychological or psychosocial nature, nine to do with the economic dimension, nine were of a social nature, and nine to do with time- and space-time related dimensions of the experience of home. Five factors were related to socioeconomic and health-related dimension.

Interestingly, most of the factors showing an effect belonged to the dimensions of the meanings and experience of home that were not related to older adults’ socioeconomic profile or health status, while these were the dimensions most commonly considered by the studies under review. The effect of thirty-one factors remains unclear, either because the types of research design in which it was identified did not concluded of the same results (effect identified or no effect identified) or because it has not been sufficiently studied using any design. Surprisingly, *mortgage and reverse mortgage*, as well as *gender* seemed to be the only factors with little effect, according to our review, but this needs to be confirmed by further studies, given the large number of female older adults and the known difference between women and men such as their respective roles in caregiving [112].

## Discussion

This systematic literature review provides an overview of the factors influencing housing decisions among older adults experiencing loss of autonomy. It shows the multiplicity of considerations involved in older adults’ housing decisions and demonstrates the strength of our theoretical framework for organizing a diversity of interdisciplinary scientific evidence. Our results lead us to make several observations. First, our results are interesting in light of the Canadian experience where the housing decisions of adults experiencing loss of autonomy are described in the research as decisions about “location of care” [113]. Professional teams helping older adults make housing choices are mostly composed of health and social service professionals (occupational therapists, nurses, physicians, social workers), and their perspective is therefore one that focuses on where the person will receive care. Our results suggest that older adults address their housing desires and the care they need to remain independent as long as possible as one and the same question. The location-of-care perspective may have led to consideration of a reduced group of factors, focusing more on the socioeconomic and health profile of older adults and on their social supports than on what else they care about in a home. The broad reach of this literature review has brought to light a diversity of other factors, suggesting that the complexity of this decision and its multidimensional nature is still underestimated [114]. Interestingly, the same perspective question came up in our decision to use the term “staying at home” as opposed to “aging in place”. The literature is not clear on the distinction between the two, as they are usually used synonymously. However, we chose to use “staying at home” because in general it reflects the perspective and preferences of the older adult himself/herself to remain in its current dwelling, while “aging in place” is a term that reflects the professional, bureaucratic or policy perspectives on the efforts to keep older people out of institutions, which could involve a move to another independent housing or not (similar to the perspective difference between the terms “housing options” vs. “location of care”). This subject of terminology choices and how they impact research would benefit from further study.

Second, to extract and analyze factors influencing the housing decision, we used the Després and Lord (2005) theoretical framework based on the meta-concept of home, designed to analyze the experiences of dwelling and neighborhood as well as the social and emotional needs of older adults [17]. This gave our analysis a new perspective and complemented the frameworks more commonly used for this purpose [11]. The diversity of factors identified showed that none of these frameworks by itself was adequate for understanding the factors that influence housing decisions. We thus created a new framework, adding a “socioeconomic profile and health” category to the Després and Lord framework for factors such as health status and age. This modified framework will allow for a fuller appreciation of the multiple dimensions of the housing decision and provide a tool for building bridges between various research domains [114]. It will also guide the updating of existing decision guides intended for older adults [115] to include consideration of the meaning and experience of home.

Third, this literature review shows a lack of diversity in studies addressing factors influencing housing decisions in old age in terms of the academic disciplines involved. More than half of the reviewed studies were written by only one author or by authors in the same research domain, while only a third benefited from experts in at least two research domains, with health sciences and social sciences being the most common combination. Even though almost 25% of the significant factors of influence were associated with the built and natural environment, less than 8% of the researchers involved in all studies were trained as geographers, planners, architects or designers. Built environment experts need to be more involved in research addressing older adults’ housing needs to contribute their knowledge about these important factors in housing decisions and provide a more complete and accurate picture of what is involved. This also highlights the importance of training researchers in architecture and urban planning [116]. A more transdisciplinary perspective is clearly needed [117–119] to inform policy and have a real impact on the quality of life of the frail elderly. However, this type of research is still rather rare and hard to finance [120].

Fourth, studies using quantitative methods focused mostly on economic, socioeconomic and health-related factors. However, quantitative methods may have a limited capacity to grasp people’s feelings, emotions and values, as well as their daily routines and social networks. Qualitative methods are more likely to be used to assess the social, psychological/psychosocial and time/space-time dimensions, as these factors are more subjective and more complex to assess using quantitative methods. Indeed, most such factors are closely linked to the meaning of home, which is the subjective heart of the housing decision. However, the effects on housing decisions of both emotional attachment to one’s dwelling and the number of years lived in the present dwelling/neighborhood remains unclear according to the results of this review, as strong quantitative studies found no effect of these factors, while strong qualitative and mixed-method studies agreed they had an important effect. While most economic and socioeconomic/health-related factors are more easily assessed with quantitative methods, studies investigating factors in the other four dimensions (psychological/psychosocial, social, time and space-time -related, built and natural environment) could also greatly benefit from more quantitative and mixed-method approaches to complement their qualitative results.

Last but not least, the effects of specific population characteristics on the housing decision, as well as several other factors identified as influential, are understudied. Very old and frail older adults were surprisingly little studied, even though we know that these are the people who suffer most autonomy loss and are most at risk of moving into long-term care [4]. Indeed, only five studies focused on very old adults and 20 specifically on frail older adults. This could be due to the difficulty of investigating this population where dementia, cognitive disorders and severe autonomy losses could limit their participation compared to younger or less frail older adults. Moreover, it may be more difficult to distinguish the very frail from the overall population of older adults, as few studies have attempted to assess frailty using validated instrument or scales and no clear definition has yet emerged in the literature [121]. Another important understudied characteristic in association with their housing decisions is the tenure status of very old adults. Only four of the 30 reviewed studies that recorded the tenure status of older adults compared the influence of being a renter or an owner on the housing decision. Yet owners and renters have been shown to have different residential mobility patterns [122, 123]. Older adults with a renter profile might move more often, and this may decrease their attachment to home, which in turn appears to be an influential factor in housing decisions. Some factors identified through this review also lack supporting scientific evidence, such as having a pet, and the experience of falls. For instance, pet ownership has been shown to have an important influence on the health of older adults in other contexts but has been mostly ignored in the context of housing decisions. The experience of falls has also been investigated in other contexts as it is a leading cause of injury-related hospitalization among older adults [124] and is the cause of most hip fractures [125], but its impact remains almost unexplored in the housing decision context. This may be because older adults seriously injured by a fall are often directly discharged into a long-term care facility [124] without having had the opportunity to participate in a proper decision-making process.

### Limitations

Our search strategy had some limitations. First, it mostly targeted databases of English-language publications. Search strategies in other languages such as French and Spanish may have found more local publications which could also have been relevant. The strategy also oriented the search results towards literature on relocation and less on staying at home. In the future, the search term “aging at home” and its synonyms could provide a broader understanding of the decision to stay at home.

Second, we did not perform all screening steps in duplicate which could have introduced a selection bias during the screening stage. However, the kappa k calculated during the pre-test suggested an excellent agreement between the two authors.

Third, the results of this review also suffer from an ethnocentric bias, as most of the studies reviewed come from Anglo-Saxon majority and higher-income countries. The proportion of these populations aged 60 years or older was greater than 20% in 2015 and is projected to be higher than 25% by 2050 [1]. This may not be surprising, as in Asian, African or Central and South American countries, for cultural reasons, families tend to keep their older relatives at home with them. Adding the perspective of other cultural approaches to housing in old age could be enlightening and suggest new housing solutions for older adults.

## Conclusion

This systematic literature review reveals the diversity of factors influencing the housing decisions of older adults. It confirms that these decisions are complex and multidimensional, and that health, safety and functional autonomy are only a few of the factors that should be considered to understand what is at stake in this type of decision and to better support older people. Important influences relate to the built environment, as well as to the social, psychological, psychosocial, spatiotemporal and decisional contexts of older adults. Several gaps in the literature were identified, mainly regarding the housing decisions of very old adults, frail older adults and the different factors that affect renters and owners.

This review also highlights the fact that this field of research is still in its infancy in terms of embracing the transdisciplinary complexity of meeting an increased demand for care and services while taking into account the importance of feeling-at-home for older adults. That said, it is surprising, albeit worrisome, that with all policies and research funding on aging put forward in the last 20 years or so, a review of scientific evidence published since 1990 on this topic has identified so few that explore decision-making about housing options, and even fewer that have identified a comprehensive collection of relevant factors. Our analysis underlines the different directions taken by each discipline and the consequences of their different methodological approaches. It brings to light the importance not only of engaging all the concerned disciplines in this field of research but of putting together multisectoral teams with complementary methodological perspectives and developing collaborative methodological approaches. Knowledge exchange is also needed so that each discipline is aware of knowledge emerging in the others. The proposed framework presented herein is a first step to bridge-building between different disciplines interested in housing decisions among older adults. Our results will guide the future development of decision guides to support healthcare professionals, older adults and their caregivers in making housing decisions.

This review emphasizes the importance of adapting dwellings and communities to older adults wishing to stay at home in the residential environment that they know and value. It also pushes us to reconsider how we design alternative housing for frail older adults. In addition to safety considerations, alternative housing should also integrate meaning-of-home considerations that could help older adults adapt to their new dwelling and rebuild their feeling of being-at-home. We hope that our results will also provide housing and healthcare professionals, policy makers, housing authorities, relocation counsellors, real estate agents and developers with the evidence they need to adopt a holistic approach in addressing the needs of older adults, not only in making housing decisions but also in providing them with alternative housing that is suitable for them.

## Supporting information

S1 Appendix Search strategy example. (DOCX)

S1 Checklist Prisma checklist. (DOC)
