# Supplementary material for: Choosing between staying at home or moving: A systematic review of factors influencing housing decisions among frail older adults
Source: PLoS One. 2018 Jan 2;13(1):e0189266. doi: 10.1371/journal.pone.0189266 (PMC5749707; doi:10.1371/journal.pone.0189266)
Supplement: S1 Appendix — (DOCX) [file pone.0189266.s001.docx]

**S1 Appendix. Search strategy example**

**AgeLine**

(older adults or “older adult”) AND (frail OR disability) AND (housing OR relocation) AND (“housing decision” OR “decision making” OR decision) AND (factors OR reasons)
NOT (dementia)

**ERIC**

(older adults or “older adult”) AND (frail OR disability) AND (housing OR relocation) AND (“housing decision” OR “decision making” OR decision) AND (factors OR reasons)

**PubMed**

(older adults or “older adult”) AND (frail OR “physical disability” OR disability) AND (housing OR relocation) AND (“housing decision” OR “decision making” OR decision) AND (factors OR reasons)

**Taylor & Francis**

(older adults or “older adult”) AND (frail OR disability) AND (housing OR relocation) AND (“housing decision” OR “decision making” OR decision) AND (factors OR reasons)
NOT (“mental disability” OR dementia) NOT (politics) NOT (drugs)

**Web of Science**

(older adults or “older adult”) AND (frail OR disability) AND (housing OR relocation) AND (“housing decision” OR “decision making” OR decision) AND (factors OR reasons)
